# Altered Cortisol Metabolism Increases Nocturnal Cortisol Bioavailability in Prepubertal Children With Type 1 Diabetes Mellitus

**DOI:** 10.3389/fendo.2021.742669

**Published:** 2021-12-14

**Authors:** Julie Brossaud, Jean-Benoît Corcuff, Vanessa Vautier, Aude Bergeron, Aurelie Valade, Anne Lienhardt, Marie-Pierre Moisan, Pascal Barat

**Affiliations:** ^1^ Nuclear Medicine, Hospital of Bordeaux, Pessac, France; ^2^ Université de Bordeaux, INRAE, Bordeaux INP, NutriNeuro, Bordeaux, France; ^3^ Pediatric Endocrinology and DiaBEA Unit, Hôpital des Enfants, Hospital of Bordeaux, Bordeaux, France; ^4^ Paediatric Unit, Hospital of Bayonne, Bayonne, France; ^5^ Paediatric Unit, Hospital of Limoges, Limoges, France

**Keywords:** 5α-Reductase, 11β-hydroxy steroid dehydrogenase-1, glucocorticoids, children, type 1 diabetes

## Abstract

**Objective:**

Disturbances in the activity of the hypothalamus-pituitary-adrenal axis could lead to functional alterations in the brain of diabetes patients. In a later perspective of investigating the link between the activity of the hypothalamus-pituitary-adrenal axis and the developing brain in children with diabetes, we assessed here nocturnal cortisol metabolism in prepubertal children with type 1 diabetes mellitus (T1DM).

**Methods:**

Prepubertal patients (aged 6–12 years) diagnosed with T1DM at least 1 year previously were recruited, along with matched controls. Nocturnal urine samples were collected, with saliva samples taken at awakening and 30 minutes after awakening. All samples were collected at home over 5 consecutive days with no detectable nocturnal hypoglycaemia. The State-Trait Anxiety Inventory (trait scale only) and Child Depression Inventory were also completed. Glucocorticoid metabolites in the urine, salivary cortisol (sF) and cortisone (sE) were measured by liquid chromatography–tandem mass spectrometry. Metabolic data were analysed by logistic regression, adjusting for sex, age, BMI and trait anxiety score.

**Results:**

Urine glucocorticoid metabolites were significantly lower in T1DM patients compared to controls. 11β-hydroxysteroid dehydrogenase type 1 activity was significantly higher, while 11β-hydroxysteroid dehydrogenase type 2, 5(α+β)-reductase and 5α-reductase levels were all lower, in T1DM patients compared to controls. There was a significant group difference in delta sE level but not in delta sF level between the time of awakening and 30 minutes thereafter.

**Conclusions:**

Our findings suggest that altered nocturnal cortisol metabolism and morning HPA axis hyperactivity in children with T1DM leads to greater cortisol bioavailability and lower cortisol production as a compensatory effect. This altered nocturnal glucocorticoid metabolism when cortisol production is physiologically reduced and this HPA axis hyperactivity question their impact on brain functioning.

## Introduction

It is well established that type 1 diabetes mellitus (T1DM) in children can have a significant impact on the developing brain, as reflected in high prevalence of depression (1) and poor performance on certain cognitive tasks (2) along with structural and functional changes (3). The mechanisms underlying depression and cognitive dysfunction in diabetic patients are complex and include factors directly related to diabetes itself, but also to diabetes-related cardiovascular disease and microvascular dysfunction (4). In children with diabetes, sustained dysregulation of blood glucose is currently considered the cause of cognitive dysfunction. However, the extent to which acute hypoglycaemia, chronic hyperglycaemia and/or blood sugar variations directly and indirectly affect brain function has yet to be clarified (2). In addition to chronic hyperglycaemia and relative insulin insufficiency, disturbances in the activity of the hypothalamus-pituitary-adrenal (HPA) axis, often implicated in autoimmune or pharmacological models of diabetes mellitus, could also participate in brain alterations (5, 6). Indeed, dysregulated glucocorticoids are well known to lead to depression (7) or mnesic dysfunctions (8).

The effect of glucocorticoids on brain function in diabetes is not only dependent on secretion but also bioavailability, which is linked with 11β-hydroxysteroid dehydrogenase (11β-HSD) cellular activity. Indeed, 11β-HSD is an important factor in peripheral cortisol metabolism. 11β-HSD is an intracellular enzyme that regulates the tissue response to cortisol at an intracellular pre-receptor step by catalyzing the interconversion of biologically active cortisol to biologically inactive cortisone. 11β-HSD type 1 (11β-HSD1) converts cortisone to cortisol predominantly in the liver and hippocampus, whereas 11β-HSD type 2 (11β-HSD2) inactivates cortisol to cortisone, mainly in the kidney, thus protecting the mineralocorticoid receptor from inappropriate stimulation by cortisol (9, 10). In animal studies, we showed that glucocorticoid levels and 11β-HSD1 activity were elevated in diabetic rats not treated with insulin. Subcutaneous administration of insulin partially prevented glucocorticoid dysregulation by decreasing 11β-HSD1 activity in the liver (11). We also showed that insulin treatment partially rescued several hippocampus-dependent behavioural and structural changes in early onset insulin-deficient diabetic rats, as well as 11β-HSD1 activity in the hippocampus (12) indicating that the elevated bioavailability of glucocorticoids may be involved in the diabetes cognitive dysfunctions.

In humans, 11β-HSD1 can be estimated from urine samples as the ratio of (alpha + beta) tetrahydrocortisol (THF) to tetrahydrocortisone (THE) (13). In a previous pilot study, we showed elevated 11β-HSD1 level in nocturnal urine samples of diabetic children (14).

The present study aims at evaluating cortisol metabolism in prepubertal children with T1DM to 1) validate these previous results, 2) widen the scope of the investigation into cortisol metabolism and 3) justify a study that will examine the association between HPA disturbances and diabetes alterations in the developing brain of T1DM children. This investigation benefitted from including diabetes patients free from micro- and macrovascular comorbidities, which may also contribute to brain alterations (15). Glucocorticoid metabolites were analysed in prepubertal children under strict conditions, focusing on repeated nocturnal excretion of glucocorticoids while also taking anxiety levels into account. Examination of nocturnal excretion of glucocorticoids, when glucocorticoid production is at its lowest due to the physiological nychthemeral cycle, should allow for the detection of subtle changes in cortisol metabolism.

## Materials and Methods

### Clinical Protocol

Prepubertal patients (aged 6–12 years) diagnosed with T1DM at least 1 year previously were routinely followed up in three different pediatric units in France (Bordeaux, Limoges and Bayonne), and enrolled in the present study. Forty percent of patients were treated by insulin pump and 60% by insulin injection. The prepubertal children in the control group were siblings of diabetic patients followed up in the 3 units. The exclusion criteria included any clinical signs of puberty onset, use of oral or inhaled corticoids during the month prior to inclusion in the study, the presence of acute infectious disease in the week prior to inclusion in the study, and any other chronic diseases apart from T1DM, including psychiatric disorders (and psychiatric treatment).

At the time of inclusion, the clinical characteristics of all participants were recorded and biological analyses were performed to confirm the absence of diabetes in the control group. Then, the State-Trait Anxiety Inventory (STAI) Trait scale and Child Depression Inventory (CDI) were completed. Only the trait scale of the STAI was used, as a measure of stable feelings of anxiety. The glucocorticoid metabolite to creatinine ratio was calculated based on nocturnal urine samples, which were taken in addition to morning salivary cortisol (sF) and cortisone (sE) samples at home over 5 consecutive days. No nocturnal hypoglycaemia (defined as glucose < 60 mg/dL) was detected at the time of awakening. Nocturnal urine samples were taken in the morning (first morning void after awakening). If hypoglycaemia occurred during the night-time or at the time of awakening, urine and saliva sampling was postponed by 24 hours. The children were asked to collect a sample of saliva to determine sF and sE using a Salivette^®^ kit (Sarstedt, Nümbrecht, Germany) upon awakening and 30 minutes thereafter. Samples were kept frozen at home until the next visit to the hospital.

The study protocol was approved by the local medical ethics committee and written informed consent was obtained from both the parents and children.

### Laboratory Measurements

Urine Glucocorticoid metabolites were measured by liquid chromatography–tandem mass spectrometry (LC-MS/MS) (ACQUITY UPLC System and TQD detector with electrospray ionization, Waters Ltd., Elstree, Hertfordshire, UK). Briefly, 6-alphamethylprednisolone was used as an internal standard, and hydrolysis with β-glucuronidase was performed before dichloromethane extraction. The ratio of each analyte to creatinine (analyte/cr) was determined.

**Figure 1:**
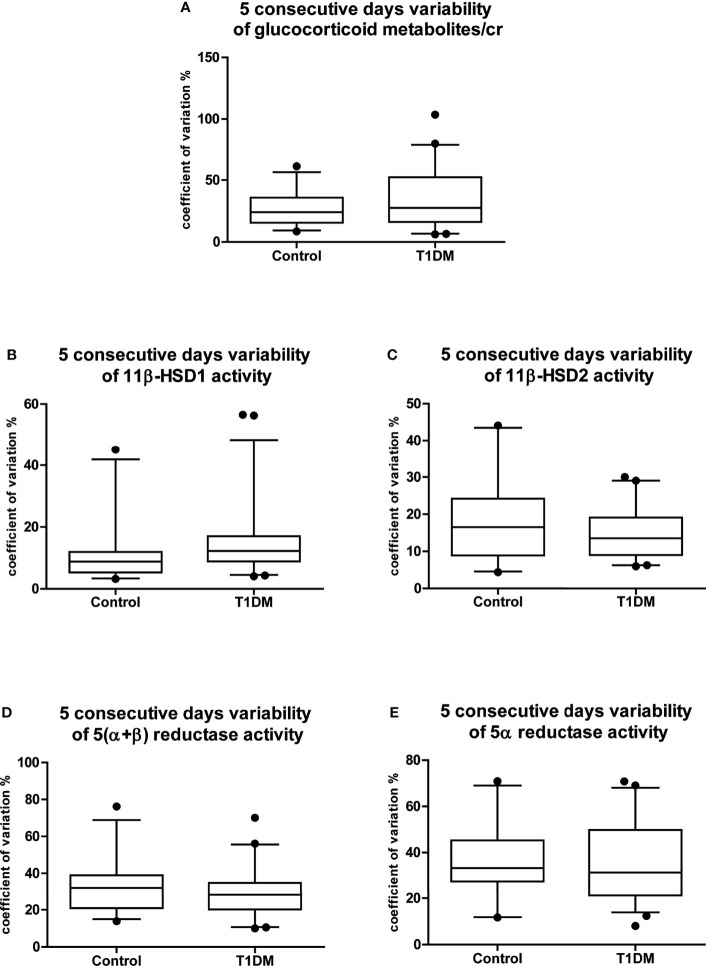
Five consecutive days variability of the main cortisol metabolism results. Coefficients of variation of the 5 consecutive days variability of **(A)** glucocorticoid metabolites/cr; **(B)** 11β-HSD1 activity; **(C)** 11β-HSD2 activity; **(D)** 5(α+β) reductase activity; **(E)** 5α reductase activity. Data are expressed as median [25-75% percentile: plot; 5-95% percentile: whiskers]. Points below and above the whiskers are drawn as individual points. T1DM, type 1 diabetes mellitus; cr, to creatinine.

Total glucocorticoid metabolites were calculated as (α+β)-THF + THE + (α+β)-cortisol + (α+β)-cortisone. Active metabolites included all metabolites derived from cortisol i.e. F+ (α+β)-THF + (α+β)-cortisol. The (α+β)-THF/THE ratio was considered a proxy for 11β-HSD1 activity. The cortisone/cortisol ratio (E/F) was considered a proxy for 11β-HSD2 activity. (α+β)-THF/F and α-THF/F were considered as proxies for 5(α+β)-reductase and 5α-reductase activity, respectively (**Figure 1**).

sF and sE was measured by LC-MS/MS (Prominence liquid chromatography system; Shimadzu, Nakagyo, Japan; and 5500 Qtrap detector, Sciex, Framingham, MA, USA). A liquefying agent (Sputasol; Thermo Fisher Scientific, Waltham, MA, USA) was added to 400 µL of saliva sample, which was then incubated for 30 minutes at 37°C. Next, solid-phase extraction with a hydrophilic lipophilic balance (Waters) was performed before injecting the extract into the LC-MS/MS system. The cortisol and cortisone concentrations were determined based on the peak area ratio of the cortisol and cortisone transitions and the Internal Standard (deuterated cortisol) transition.

### Statistical Analysis

Analyses and graphs were performed using SAS (ver. 9.4; SAS Institute, Cary, NC, USA), R (R Development Core Team, Vienna, Austria), STATISTICA 6 (TIBCO Software, Palo Alto, CA, USA) and GraphPad Prism software.

Unless otherwise stated, all values are presented as the mean ± standard deviation and *p* < 0.05 was considered significant. The significance of the differences in qualitative variables between T1DM patients and controls was tested using a logistic regression model. The correlation within diabetic and non-diabetic “pairs” (siblings of the subjects) has been taken into account by a pair-related random effect.

Dispersion was estimated by the coefficient of variation. The homogeneity of variance was assessed using Levene’s test; the variance in the data for all metabolites did not differ between controls and T1DM patients (**Figures 1A**–**E**). The intraday variability was also similar between the groups for all metabolites; thus, results are reported as the mean of five samples for all of the children.

Group differences in metabolic data were evaluated using a logistic regression model adjusted for sex, age, BMI and STAI score. Mean and 95% confidential intervals are provided. Pearson correlation analyses were conducted to identify associations among age, BMI, insulin dose, HbA1c and metabolic data.

## Results

### Description of the Study Population

The baseline characteristics of the T1DM (n = 49) and control (n = 26) groups are presented in **Table 1**. No significant differences in age, sex, education level or clinical characteristics such as weight, height, BMI, waist circumference, systolic or diastolic blood pressure or Tanner score were observed between the groups. All children were pre-pubertal. However, the STAI Trait scale score tended to be higher in the control group (*p* = 0.07). No difference was observed between the two groups in CDI score.

**Table 1:** Characteristics of the study population at enrolment.

	Controls (n = 26)	T1DM (n = 49)	p
**Age (years)**	9.0 (1.7)	9.3 (1.4)	0.41
**Sex (male/female)**	12/14	27/22	0.46
**Education level relative to children of the same age (%)**	100	95.9	0.29
**Onset of diabetes (years)**		3.7 (2.2)	
**Height (cm)**	133.5 (9.3)	132.4 (10.9)	0.66
**Weight (kg)**	28.8 (6.3)	28.9 (5.7)	0.94
**BMI (kg/m²)**	16.0 (1.7)	16.3 (1.3)	0.39
**Systolic blood pressure (mmHg)**	107.0 (9.3)	104.0 (9.0)	0.17
**Diastolic blood pressure (mmHg)**	64.9 (7.1)	62.0 (6.5)	0.07
**Tanner stage**			0.94
**B1 or G1 (%)**	100	100	
**P1 (%)**	96.2	95.9	
**P2 (%)**	3.8	4.1	
**Insulin dose (UI/day)**		23.5 (9.5)	
**Glycaemia (g/l)**	0.8 (0.1)		
**HbA1c (%)**	5.3 (0.3)	7.6 (0.7)	< 0.0001
**Child Depression Inventory score**	9.0 (6.3)	6.9 (6.0)	0.16
**STAI score**	33.0 (7.8)	29.7 (6.6)	0.057

Results are expressed as mean (SD). CDI, Child Depression Inventory; STAI, State-Trait Anxiety Inventory (trait scale only).

### Urine Glucocorticoid Metabolites in T1DM Children

No differences were observed between males and females in levels of urine glucocorticoid metabolites. The data for each group before and after adjustment for the STAI Trait scale score and urine cortisol and cortisone metabolites, as well as 11β-HSD1, 11β-HSD2, 5(α+β)-reductase and 5α-reductase enzymatic activity, are shown in **Table 2**.

**Table 2:** Metabolites of cortisol and cortisone of the T1DM and control groups.

Metabolite	Group	Group effect	Group effect adjusted for STAI score
**F/cr** (µg/mmol)	Control	4.5 [3.8;5.3]	4.7 [3.8;5.7]
	T1DM	5.2 [4.6;5.8]	5.3 [4.6;6.1]
**E/cr** (µg/mmol)	Control	11.1 [9.5;12.6]	11.0 [9.1;12.8]
	T1DM	11.2 [10.0;12.3]	11.3 [9.9;12.7]
**β-THF/cr** (µg/mmol)	Control	111 [98;127]	107 [89;125]
	T1DM	95 [84;107]	91 [78;105]
**α-THF/cr** (µg/mmol)	Control	30.4 [25.9;34.8]	30.3 [25.2;35.4]
	T1DM	25.3 [22.0;28.7]	24.2 [20.3;28.1]
**THE/cr** (µg/mmol)	Control	355 [317;392]***	338 [293;383]***
	T1DM	256 [227;284]	261 [227;296]
**α-cortol/cr** (µg/mmol)	Control	9.22 [7.41;11.03]	9.26 [7.76;10.76]*
	T1DM	11.3 [9.98;12.71]	10.8 [9.71;11.99]
**β-cortol/cr** (µg/mmol)	Control	24.6 [20.7:28.5]**	23.0 [18.7:27.4]**
	T1DM	25.6 [22.6;28.5]	25.7 [22.4;29.0]
**α-cortolone/cr** (µg/mmol)	Control	79.7 [70.1;89.4]	75.9 [65.2;86.5]
	T1DM	78.8 [71.5;86.1]	81.2 [73.1;89.3]
**β-cortolone/cr** (µg/mmol)	Control	58.8 [52.1;65.5]	54.5 [47.0;62.0]
	T1DM	45.9 [40.8;50.9]	47.9 [42.2;53.6]
**Total cortisol metabolites/cr** (µg/mmol)	Control	173 [146;200]	175 [152;198]
	T1DM	146 [127;166]	157 [140;175]
**Total glucocorticoid metabolites/cr** (µg/mmol)	Control	676 [606;745]*	655 [576;734]*
	T1DM	564 [513;616]	560 [500;620]
**(α+β)-THF/THE** **Reflecting 11β-HSD1 activity**	Control	0.42 [0.38;0.46]*	0.42 [0.37;0.47]*
	T1DM	0.48 [0.45;0.51]	0.47 [0.43;0.50]
**E/F** **Reflecting 11β-HSD2 activity**	Control	2.53 [2.26;2.89]*	2.38 [2.08;2.77]*
	T1DM	2.17 [2.01;2.35]	2.07 [1.89;2.28]
**(α+β)-THF/F** **Reflecting 5(α+β) reductase activity**	Control	37.4 [32.7;42.1]**	34.5 [29.4;39.7]**
	T1DM	28.2 [24.7;31.8]	26.3 [22.4;30.2]
**α-THF/F** **Reflecting 5α reductase activity**	Control	8.11 [6.95;9.27]***	7.62 [6.43;8.81]**
	T1DM	5.62 [4.76;6.47]	5.32 [4.42;6.22]
**sF T0** (nmol/l)	Control	3.65 [3.02;4.28]	3.61 [3.02;4.37]
	T1DM	3.51 [3.03;3.98]	3.59 [2.81;4.21]
**sF T30** (nmol/l)	Control	4.16 [3.38;4.94]	4.36 [3.43;5.28]
	T1DM	4.49 [3.92;5.05]	4.58 [3.88;5.28]
**sF Delta T30-T0** (nmol/l)	Control	0.30 [-0.54;1.13]	0.71 [0.34;1.66]
	T1DM	1.14 [0.52;1.76]	1.00 [-0.15;1.57]
**sE T0** (nmol/l)	Control	15.3 [13.5;17.0]	15.0 [14.7;15.3]
	T1DM	13.9 [12.5;16.0]	13.9 [13.7;14.2]
**sE T30** (nmol/l)	Control	16.0 [14.4;19.1]	16.7 [16.4;17.1]
	T1DM	17.8 [16.0;19.5]	17.4 [17.1;17.7]
**sE Delta T30-T0** (nmol/l)	Control	1.42 [-0.66;3.50]*	1.63 [1.30;1.96]*
	T1DM	4.42 [2.82;6.07]	4.21 [3.93;4.48]
**sE/sF T0 ratio**	Control	5.23 [4.34;6.13]	5.05 [4.91;5.18]
	T1DM	4.68 [4.17;5.19]	4.73 [4.64;4.82]
**sE/sF T30 ratio**	Control	4.99 [4.19;5.79]	4.88 [4.76;5.00]
	T1DM	4.50 [4.03;4.96]	4.58 [4.50;4.67]

Group differences in metabolic data were evaluated with a logistic regression model, adjusted for the STAI trait scale score. Data are expressed as mean [95% confidential interval].

T1DM, type 1 diabetes mellitus; F, cortisol; E, cortisone; THF, tetrahydrocortisol; THE, tetrahydrocortisone; sF, salivary cortisol; sE, salivary cortisone;/cr, to creatinine; STAI, State-Trait Anxiety Inventory (trait scale only). *p < 0.05; **p < 0.01; ***p < 0.001.

**Figure 2:**
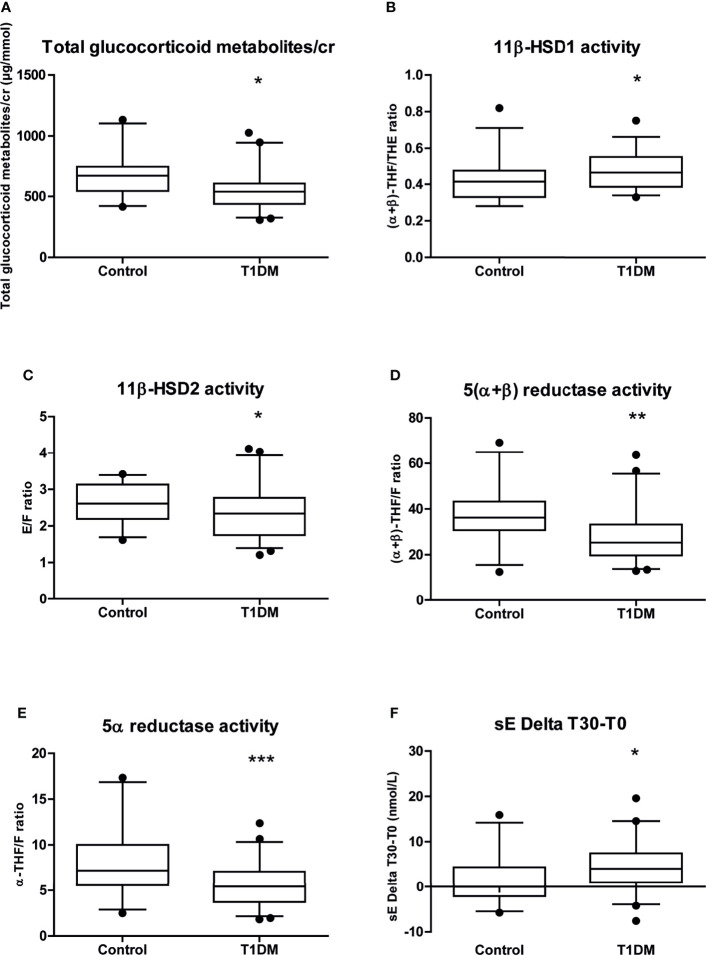
Whisker plots representation of the main cortisol metabolism results from the data in **Table 2**. Whisker plots of **(A)** glucocorticoid metabolites/cr; **(B)** 11β-HSD1 activity; **(C)** 11β-HSD2 activity; **(D)** 5(α+β) reductase activity; **(E)** 5α reductase activity; **(F)** sE Delta T30-T0. Data are expressed as median [25-75% percentile: plot; 5-95% percentile: whiskers]. Points below and above the whiskers are drawn as individual points. T1DM, type 1 diabetes mellitus; F, cortisol; E, cortisone; THF, tetrahydrocortisol; THE, tetrahydrocortisone; sF, salivary cortisol; sE, salivary cortisone;/cr, to creatinine. *p < 0.05; **p < 0.01; ***p < 0.001.

Due to the lower THE/cr ratio, the total glucocorticoid metabolite level was significantly lower in T1DM patients compared to controls. The 11β-HSD1 activity was significantly higher, while 11β-HSD2, 5(α+β)-reductase and 5α-reductase activities were significantly lower, in T1DM patients compared to controls (**Figures 2A**–**E**). These differences remained after adjusting the regression analyses for STAI trait anxiety score.

### Salivary Cortisol and Cortisone on Awakening

No difference was observed between males and females in sF and sE levels. There was also no significant difference between the T1DM patients and controls in sF and sE level on awakening (sF T0 or sE T0) or 30 minutes thereafter (sF T30 or sE T30) neither in sE/sF ratio level, including after adjusting the analyses for STAI trait anxiety score. However, we found a significant difference in delta sE levels between the time of awakening and 30 minutes thereafter (sE Delta T30-T0) (**Table 2** and **Figure 2F**).

### Correlation Between Metabolic Data and Clinical Characteristics

No significant correlation was found between 11β-HSD1 or 5α-reductase activity and clinical characteristics: neither 11β-HSD1 nor 5α-reductase activity was significantly correlated with BMI in the T1DM or control group, or with HbA1c or insulin levels in the T1DM group.

## Discussion

Our analysis of the nocturnal urine samples of prepubertal children, obtained over a 5-day period in the home setting, revealed altered nocturnal cortisol metabolism (higher 11β-HSD1 and lower 11β-HSD2 activity) in those with T1DM, in addition to lower glucocorticoid metabolite excretion and 5α-reductase activity. The results remained significant after adjusting for STAI trait scale score. In addition to these changes in nocturnal glucocorticoid metabolism, the morning reactivity of the HPA axis, as indexed by sE Delta T30-T0 levels upon awakening, was significantly higher in the T1DM group than in the control group.

To later assess brain alterations in T1DM patients, we specifically targeted prepubertal children due to the absence of micro- and macro-vascular comorbidities in that population. Our results call into question sexual dimorphism in cortisol metabolism in this age group. Finken et al. described sexual dimorphism in cortisol metabolism in association with 5α-reductase, but not 11β-HSD1, activity in healthy young adults (16). In healthy children, the 24-hour excretion rate of glucocorticoid metabolites rose markedly between the ages of 4 and 14 years in both boys and girls, in association with the body fat percentage and BMI (17). No sex difference in glucocorticoid metabolite excretion, or 11β-HSD1 and 11β-HSD2 activities, was seen in healthy prepubertal children, unlike pubertal children (17). Similarly, the present study found no sex difference in the glucocorticoid metabolite excretion rate or enzymatic activity, in either the T1DM or control group, from which individuals with any signs of pubertal onset were excluded.

In a previous study, 11β-HSD2 and 11β-HSD1 activities measured in 24 hour urine samples were similarly elevated in children aged below 10 years diagnosed with T1DM (18) while others found a decrease of 11β-HSD1 activity but in adults (19). In our study, we confirmed our previous finding of elevated 11β-HSD1 level in children with T1DM, with urine samples collected over the course of a single night (14). However, we also found that 11β-HSD2 activity was lower during the night. Our results underline the importance of measuring subtle nocturnal changes in glucocorticoid metabolism. The circadian rhythm of cortisol secretion dictates that the rate of production of cortisol metabolites at night is low. Changes in nocturnal 11β-HSD type 1 and 2 activity suggest an abnormal increase in cortisol bioavailability due to upregulated conversion of cortisone to cortisol.

5α-reductase is responsible for the irreversible reduction of cortisol to 5α-THF, which mainly occurs in the liver and contributes to the clearance of cortisol. Decreased production and metabolic clearance of cortisol, as determined by 24-hour urine collection, has previously been described in normotensive type 1 diabetic males with adequate glycaemic control and without severe complications (9), as well as in children aged below 10 years with T1DM. In our study, reductase activity was decreased, mainly due to a lower level of 5α-reductase activity. Furthermore, we confirmed diminished glucocorticoid production, based on the total urinary glucocorticoid metabolite excretion. The impaired production and metabolic clearance of cortisol are hypothesized to be due to lower 5α-reductase activity, which could result in reduced metabolic clearance of cortisol. Due to the comparatively longer half-life of cortisol, this could in turn lead to a decrease in cortisol production *via* a negative feedback loop (9).

Children with T1DM show relative insulin insufficiency in the liver on subcutaneous delivery of insulin and, as a consequence, the absence of a first hepatic pass. Higher 11β-HSD1 activity and lower 5α-reductase activity could result from this insulin insufficiency. Regarding 11β-HSD1 activity, this hypothesis (proposed by Kerstens et al.) was rejected because 11β-HSD1 activity increased in healthy adults submitted to a hyperinsulinaemic euglycaemic clamp (20). However, in a rodent model of insulin-deficient diabetes, we showed that intraperitoneal insulin led to a decrease in 11β-HSD1 activity in the liver, whereas subcutaneous insulin did not (11). At the cellular level, results are contradictory regarding whether glucose control (via insulin administration) has a direct (21) or indirect inhibitory effect (22) on 11β-HSD1 transcription or activity.

The activity of 5α-reductase was shown to be increased in association with hyperinsulinemia in patients with polycystic ovary syndrome (23), as well as in those with type 2 diabetes (24) or impaired glucose tolerance (25). Furthermore, Kayampilly et al. reported dose-dependent stimulation of 5α-reductase activity by insulin in a human granulosa cell line (26). In the normal-weight children dependent on subcutaneous insulin treatment included in our study, we speculate that relative insulin deficiency was responsible for the observed increase in 11β-HSD1 activity and decrease in 5α-reductase activity, which both contribute to greater cortisol bioavailability and suppression of cortisol production as a compensatory effect.

Hyperactivity of the HPA axis has been well described in adults with insulin-dependent diabetes (27, 28). In the present study, we investigated HPA activity *via* repeated saliva sampling in the home setting. We found a significant difference between the T1DM and control groups in levels of sE Delta T30-T0, thus indicating an increase in HPA axis reactivity. Indeed as 11β-HSD2 is very active in saliva, cortisol is immediately converted in cortisone which thus reflects HPA axis reactivity when cortisol production is at its highest level (i.e. during the nychthemeral cycle). High intragroup variability was seen in the sF and sE data; we speculate that this was due to the difficulty that the children had in following the instructions as they pertained to taking repeated measurements (where the samples must be obtained on awakening and exactly 30 minutes thereafter). Furthermore, when interpreting our data suggesting reactivity of the HPA axis, changes in 5α-reductase activity should be taken account. Indeed, due to the longer half-life of corticosterone, mice deficient in 5α-reductase type 1 show an impaired adrenal response to adrenocorticotropic hormone (ACTH) (29).

For ethical and practical reasons, we used the siblings of diabetic children as the control subjects. Previous studies have shown a reassuring psychosocial effect (30) and no adverse psychiatric disorders (31) in siblings of children with T1DM. Because HPA axis activity could be associated with chronic stress, anxiety or depression we measured these traits in our cohort. Unexpectedly, the control group tended to show higher STAI trait scale scores than the T1DM patients, but there was no significant group difference in depression. Although we focused on trait rather than state anxiety, it is possible that exposing the siblings of the diabetic children to a hospital environment, to which they are not accustomed, may have influenced our findings. Finally, adjusting the analysis for STAI trait score had little impact on the urinary metabolite data, but was useful in confirming the absence of any differences between the two groups in terms of the activity and reactivity of the HPA axis.

One limitation of this study was that nocturnal hypoglycaemia was not detected using a glucose sensor. However, the parents were asked not to take urine samples during periods of nocturnal or waking hypoglycaemia. Samples were obtained over a 5-day period in all children, and differences in the variability of metabolic data were not apparent between the two groups; therefore, we used the mean values of these consecutively obtained samples in the analysis. Furthermore, our results suggested lower nocturnal production of cortisol in children with T1DM. For these reasons, it is unlikely that clinically significant nocturnal hypoglycaemia can explain our findings. Another limitation of this study is that only morning sF and sE have been sampled. Nocturnal sF and sE at bedtime would be interesting measurements to underline changes in cortisol negative feedback (32), even though bedtime is variable between children.

In conclusion, our findings suggested that altered nocturnal cortisol metabolism in children with T1DM leads to greater cortisol bioavailability due to an abnormal increase in the conversion of cortisone to cortisol, which ultimately lowers cortisol production as a compensatory effect. Moreover, we objectified an increase of the morning HPA axis reactivity. Overall, this greater availability of cortisol when cortisol production is physiologically reduced due to the circadian rhythm of cortisol and this higher HPA axis reactivity prompt many questions regarding its impact on the brain. It is well known that the activity of the HPA axis and glucocorticoid production have an impact on brain structures involved in cognition and mental health (33), specifically in the context of diabetes (34). Furthermore, our results call into question the route of insulin administration, and its ability to prevent altered glucocorticoid metabolism in T1DM patients. A recent rat study of early onset insulin-deficient diabetes supported this hypothesis, by showing that subcutaneous insulin treatment cannot completely prevent several of the hippocampal-dependent behavioural and structural alterations linked with an increase in local 11β-HSD1 activity (12). It also suggested that an increase in peripheral glucocorticoid bioavailability may be associated with a local increase in these hormones within the hippocampus, which has central consequences. Thus, our findings suggest that elevated 11β-HSD1 activity and lower 5α-reductase activity should be considered as a potential factor for cortisol-dependent brain alterations in diabetic patients.

## Author’s Note

The English in this document has been checked by at least two professional editors, both native speakers of English. For a certificate, please see: (http://www.textcheck.com/certificate/qiFziq).

## Data Availability Statement

The raw data supporting the conclusions of this article will be made available by the authors, without undue reservation.

## Ethics Statement

The studies involving human participants were reviewed and approved by Le comité de protection des personnes (CPP). Written informed consent to participate in this study was provided by the participants’ legal guardian/next of kin.

## Author Contributions

JB, J-BC, VV, AB, AV, AL, and PB performed the research. JB, J-BC, M-PM, and PB designed the research study. JB, J-BC, and M-PM contributed essential reagents or tools. JB, J-BC, M-PM, and PB analyzed the data. JB, J-BC, M-PM, and PB wrote the paper. All authors contributed to the article and approved the submitted version.

## Funding

This work was supported by the “Programme Hospitalier de Recherche Clinique” CHUBX 2011/24 of Bordeaux’ Hospital.

## Conflict of Interest

The authors declare that the research was conducted in the absence of any commercial or financial relationships that could be construed as a potential conflict of interest.

## Publisher’s Note

All claims expressed in this article are solely those of the authors and do not necessarily represent those of their affiliated organizations, or those of the publisher, the editors and the reviewers. Any product that may be evaluated in this article, or claim that may be made by its manufacturer, is not guaranteed or endorsed by the publisher.

## References

[1] Zanoveli JM Morais H Dias IC Schreiber AK Souza CP Cunha JM. Depression Associated With Diabetes: From Pathophysiology to Treatment. Curr Diabetes Rev (2016) 12(3):165–78. doi: 10.2174/1573399811666150515125349 25981499

[2] Litmanovitch E Geva R Rachmiel M. Short and Long Term Neuro-Behavioral Alterations in Type 1 Diabetes Mellitus Pediatric Population. World J Diabetes (2015) 6(2):259–70. doi: 10.4239/wjd.v6.i2.259 PMC4360419 25789107

[3] Biessels GJ Reijmer YD. Brain Changes Underlying Cognitive Dysfunction in Diabetes: What can We Learn From MRI? Diabetes (2014) 63(7):2244–52. doi: 10.2337/db14-0348 24931032

[4] Shalimova A Graff B Gasecki D Wolf J Sabisz A Szurowska E. Cognitive Dysfunction in Type 1 Diabetes Mellitus. J Clin Endocrinol Metab (2019) 104(6):2239–49. doi: 10.1210/jc.2018-01315 30657922

[5] Beauquis J Homo-Delarche F Revsin Y De Nicola AF Saravia F. Brain Alterations in Autoimmune and Pharmacological Models of Diabetes Mellitus: Focus on Hypothalamic-Pituitary-Adrenocortical Axis Disturbances. Neuroimmunomodulation (2008) 15(1):61–7. doi: 10.1159/000135625 18667801

[6] MacLullich AM Seckl JR. Diabetes and Cognitive Decline: Are Steroids the Missing Link? Cell Metab (2008) 7(4):286–7. doi: 10.1016/j.cmet.2008.03.012 18396133

[7] Menke A. Is the HPA Axis as Target for Depression Outdated, or Is There a New Hope? Front Psychiatry (2019) 10:101. doi: 10.3389/fpsyt.2019.00101 30890970 PMC6413696

[8] de Quervain D Schwabe L Roozendaal B. Stress, Glucocorticoids and Memory: Implications for Treating Fear-Related Disorders. Nat Rev Neurosci (2017) 18(1):7–19. doi: 10.1038/nrn.2016.155 27881856

[9] Kerstens MN Luik PT van der Kleij FG Boonstra AH Breukelman H Sluiter WJ. Decreased Cortisol Production in Male Type 1 Diabetic Patients. Eur J Clin Invest (2003) 33(7):589–94. doi: 10.1046/j.1365-2362.2003.01171.x 12814396

[10] Chapman K Holmes M Seckl J. 11beta-Hydroxysteroid Dehydrogenases: Intracellular Gate-Keepers of Tissue Glucocorticoid Action. Physiol Rev (2013) 93(3):1139–206. doi: 10.1152/physrev.00020.2012 PMC3962546 23899562

[11] Rougeon V Moisan MP Barthe N Beauvieux MC Helbling JC Pallet V. Diabetes and Insulin Injection Modalities: Effects on Hepatic and Hippocampal Expression of 11beta-Hydroxysteroid Dehydrogenase Type 1 in Juvenile Diabetic Male Rats. Front Endocrinol (Lausanne) (2017) 8:81. doi: 10.3389/fendo.2017.00081 28458655 PMC5394469

[12] Marissal-Arvy N Campas MN Semont A Ducroix-Crepy C Beauvieux MC Brossaud J. Insulin Treatment Partially Prevents Cognitive and Hippocampal Alterations as Well as Glucocorticoid Dysregulation in Early-Onset Insulin-Deficient Diabetic Rats. Psychoneuroendocrinology (2018) 93:72–81. doi: 10.1016/j.psyneuen.2018.04.016 29702445

[13] Walker BR Andrew R. Tissue Production of Cortisol by 11beta-Hydroxysteroid Dehydrogenase Type 1 and Metabolic Disease. Ann N Y Acad Sci (2006) 1083:165–84. doi: 10.1196/annals.1367.012 17148739

[14] Barat P Brossaud J Lacoste A Vautier V Nacka F Moisan MP. Nocturnal Activity of 11beta-Hydroxy Steroid Dehydrogenase Type 1 Is Increased in Type 1 Diabetic Children. Diabetes Metab (2013) 39(2):163–8. doi: 10.1016/j.diabet.2012.10.001 23159804

[15] Biessels GJ Deary IJ Ryan CM. Cognition and Diabetes: A Lifespan Perspective. Lancet Neurol (2008) 7(2):184–90. doi: 10.1016/S1474-4422(08)70021-8 18207116

[16] Finken MJ Andrews RC Andrew R Walker BR. Cortisol Metabolism in Healthy Young Adults: Sexual Dimorphism in Activities of A-Ring Reductases, But Not 11beta-Hydroxysteroid Dehydrogenases. J Clin Endocrinol Metab (1999) 84(9):3316–21. 10.1210/jcem.84.9.6009 10487705

[17] Dimitriou T Maser-Gluth C Remer T. Adrenocortical Activity in Healthy Children Is Associated With Fat Mass. AmJClinNutr (2003) 77(3):731–6. doi: 10.1093/ajcn/77.3.731 12600869

[18] Remer T Maser-Gluth C Boye KR Hartmann MF Heinze E Wudy SA. Exaggerated Adrenarche and Altered Cortisol Metabolism in Type 1 Diabetic Children. Steroids (2006) 71(7):591–8. doi: 10.1016/j.steroids.2006.02.005 16616286

[19] Dullaart RP Ubels FL Hoogenberg K Smit AJ Pratt JJ Muntinga JH. Alterations in Cortisol Metabolism in Insulin-Dependent Diabetes Mellitus: Relationship With Metabolic Control and Estimated Blood Volume and Effect of Angiotensin-Converting Enzyme Inhibition. J Clin Endocrinol Metab (1995) 80(10):3002–8. doi: 10.1210/jcem.80.10.7559888 7559888

[20] Kerstens MN Riemens SC Sluiter WJ Pratt JJ Wolthers BG Dullaart RP. Lack of Relationship Between 11beta-Hydroxysteroid Dehydrogenase Setpoint and Insulin Sensitivity in the Basal State and After 24h of Insulin Infusion in Healthy Subjects and Type 2 Diabetic Patients. Clin Endocrinol (Oxf) (2000) 52(4):403–11. doi: 10.1046/j.1365-2265.2000.00975.x 10762282

[21] Voice MW Seckl JR Edwards CR Chapman KE. 11 Beta-Hydroxysteroid Dehydrogenase Type 1 Expression in 2S FAZA Hepatoma Cells Is Hormonally Regulated: A Model System for the Study of Hepatic Glucocorticoid Metabolism. Biochem J (1996) 317( Pt 2):621–5. 10.1042/bj3170621 PMC1217531 8713094

[22] Fan Z Du H Zhang M Meng Z Chen L Liu Y. Direct Regulation of Glucose and Not Insulin on Hepatic Hexose-6-Phosphate Dehydrogenase and 11beta-Hydroxysteroid Dehydrogenase Type 1. Mol Cell Endocrinol (2011) 333(1):62–9. doi: 10.1016/j.mce.2010.12.010 PMC3741409 21163329

[23] Tsilchorozidou T Honour JW Conway GS. Altered Cortisol Metabolism in Polycystic Ovary Syndrome: Insulin Enhances 5{Alpha}-Reduction But Not the Elevated Adrenal Steroid Production Rates. J Clin Endocrinol Metab (2003) 88(12):5907–13. doi: 10.1210/jc.2003-030240 14671189

[24] Andrews RC Herlihy O Livingstone DE Andrew R Walker BR. Abnormal Cortisol Metabolism and Tissue Sensitivity to Cortisol in Patients With Glucose Intolerance. J Clin Endocrinol Metab (2002) 87(12):5587–93. doi: 10.1210/jc.2002-020048 12466357

[25] Tomlinson JW Finney J Gay C Hughes BA Hughes SV Stewart PM. Impaired Glucose Tolerance and Insulin Resistance Are Associated With Increased Adipose 11beta-Hydroxysteroid Dehydrogenase Type 1 Expression and Elevated Hepatic 5alpha-Reductase Activity. Diabetes (2008) 57(10):2652–60. doi: 10.2337/db08-0495 PMC2551674 18633104

[26] Kayampilly PP Wanamaker BL Stewart JA Wagner CL Menon KM. Stimulatory Effect of Insulin on 5alpha-Reductase Type 1 (SRD5A1) Expression Through an Akt-Dependent Pathway in Ovarian Granulosa Cells. Endocrinology (2010) 151(10):5030–7. doi: 10.1210/en.2010-0444 PMC2946143 20810561

[27] Roy M Collier B Roy A. Hypothalamic-Pituitary-Adrenal Axis Dysregulation Among Diabetic Outpatients. Psychiatry Res (1990) 31(1):31–7. doi: 10.1016/0165-1781(90)90106-F 2156275

[28] Roy MS Roy A Gallucci WT Collier B Young K Kamilaris TC. The Ovine Corticotropin-Releasing Hormone-Stimulation Test in Type I Diabetic Patients and Controls: Suggestion of Mild Chronic Hypercortisolism. Metabolism (1993) 42(6):696–700. doi: 10.1016/0026-0495(93)90235-G 8389960

[29] Livingstone DE Di Rollo EM Yang C Codrington LE Mathews JA Kara M. Relative Adrenal Insufficiency in Mice Deficient in 5alpha-Reductase 1. J Endocrinol (2014) 222(2):257–66. doi: 10.1530/JOE-13-0563 PMC4104038 24872577

[30] Jackson C Richer J Edge JA. Sibling Psychological Adjustment to Type 1 Diabetes Mellitus. Pediatr Diabetes (2008) 9(4 Pt 1):308–11. doi: 10.1111/j.1399-5448.2008.00385.x 18466212

[31] Butwicka A Frisen L Almqvist C Zethelius B Lichtenstein P. Risks of Psychiatric Disorders and Suicide Attempts in Children and Adolescents With Type 1 Diabetes: A Population-Based Cohort Study. Diabetes Care (2015) 38(3):453–9. doi: 10.2337/dc14-0262 PMC4338504 25650362

[32] Raff H Phillips JM. Bedtime Salivary Cortisol and Cortisone by LC-MS/MS in Healthy Adult Subjects: Evaluation of Sampling Time. J Endocr Soc (2019) 3(8):1631–40. doi: 10.1210/js.2019-00186 PMC6682408 31403090

[33] Lupien SJ McEwen BS Gunnar MR Heim C. Effects of Stress Throughout the Lifespan on the Brain, Behaviour and Cognition. NatRevNeurosci (2009) 10(6):434–45. doi: 10.1038/nrn2639 19401723

[34] Stranahan AM Arumugam TV Cutler RG Lee K Egan JM Mattson MP. Diabetes Impairs Hippocampal Function Through Glucocorticoid-Mediated Effects on New and Mature Neurons. NatNeurosci (2008) 11(3):309–17. doi: 10.1038/nn2055 PMC2927988 18278039

